# Comparative GC-MS Analysis of Fresh and Dried Curcuma Essential Oils with Insights into Their Antioxidant and Enzyme Inhibitory Activities

**DOI:** 10.3390/plants12091785

**Published:** 2023-04-27

**Authors:** Nouran M. Fahmy, Shaimaa Fayez, Abdullahi Ibrahim Uba, Mohammad Ali Shariati, Abdullah S. M. Aljohani, Ibrahim M. El-Ashmawy, Gaber El-Saber Batiha, Omayma A. Eldahshan, Abdel Nasser Singab, Gokhan Zengin

**Affiliations:** 1 Department of Pharmacognosy, Faculty of Pharmacy, Ain Shams University, Cairo 11566, Egypt; 2 Department of Molecular Biology and Genetics, Istanbul AREL University, Istanbul 34537, Türkiye; 3 Semey Branch of the Institute, Kazakh Research Institute of Processing and Food Industry, 238«G» Gagarin Ave., Almaty 050060, Kazakhstan; 4 Department of Veterinary Medicine, College of Agriculture and Veterinary Medicine, Qassim University, Buraydah 51452, Saudi Arabia; 5 Pharmacology Department, Faculty of Veterinary Medicine, Alexandria University, Alexandria 22758, Egypt; 6 Department of Pharmacology and Therapeutics, Faculty of Veterinary Medicine, Damanhour University, Damanhour 22511, Egypt; 7 Center for Drug Discovery Research and Development, Ain Shams University, Cairo 11566, Egypt; 8 Department of Biology, Science Faculty, Selcuk University, Konya 42130, Türkiye

**Keywords:** turmeric essential oil, fresh vs. dried, GC-MS, antioxidant, enzyme inhibition, docking, multivariate analysis, molecular dynamics

## Abstract

Species belonging to the Zingiberaceae family are of high nutritional, industrial, and medicinal values. In this study, we investigated the effect of processing steps (fresh vs. dried milled rhizomes) and extraction methodologies (hydrodistillation vs. hexane extraction) of curcuma essential oil on its chemical content (using GC-MS analysis), its antioxidant behavior (using in vitro assays such as DPPH, ABTS, CUPRAC, FRAP, phosphomolybdenum, and metal chelation), and its enzyme inhibitory activities (on tyrosinase, acetylcholinesterase, butylcholinesterase, α-amylase, and α-glucosidase) supported by multivariate analysis, in silico studies, and molecular dynamics. The GC-MS investigations revealed a high degree of similarity in the chemical profile of fresh hydrodistilled and hexane-extracted essential oils with tumerone and curlone being the major metabolites. The extraction techniques affected the concentrations of other minor constituents such as terpinolene, caryophylla-4(12), 8(13)-dien-5α-ol, and neo-intermedeol, which were almost exclusively detected in the hydrodistilled fresh essential oil; however, zingiberene and β-sesquiphellandrene were predominant in the hexane-extracted fresh essential oil. In the dried curcuma rhizomes, tumerone and curlone contents were significantly reduced, with the former being detected only in the hydrodistilled essential oil while the latter was doubly concentrated in the hexane-derived oil. Constituents such as D-limonene and caryophyllene oxide represented ca. 29% of the dried hydrodistilled essential oil, while ar-turmerone was detected only in the dried hydrodistilled and hexane-extracted essential oils, representing ca. 16% and 26% of the essential oil composition, respectively. These variations in the essential oil chemical content have subsequently affected its antioxidant properties and enzyme inhibitory activities. In silico investigations showed that hydrophobic interactions and hydrogen bonding were the characteristic binding modes of the bioactive metabolites to their respective targets. Molecular dynamics revealed the stability of the ligand-target complex over time. From the current study we conclude that fresh hexane-extracted essential oil showed the best radical scavenging properties, and fresh rhizomes in general display better enzyme inhibitory activity regardless of the extraction technique.

## 1. Introduction

Species of the Zingiberaceae (ca. 1500), such as curcuma, ginger, and galangal, are of great medicinal, culinary (i.e., condiments), and economical (i.e., dyes, fragrances, and natural food colorants) relevance with particular distribution in the tropics and subtropics, especially in Asia, which harbors the highest number of taxa [1,2]. Curcuma (turmeric—golden spice) is among the largest genera in the Zingiberaceae family, comprising over 80 species [3]. Its ground rhizomes have important medicinal values owing to its content of curcuminoids, flavonoids, phenolic acids, and essential oil, which explains its worldwide recognition as a functional food [4]. Curcuminoid pigments (curcumin, demethoxycurcumin, bisdemethoxycurcumin) and turmeric essential oil constitute ca. 30–45% and 15–20% of the total secondary metabolites produced by the plant, respectively [5,6]. 

Harvest and postharvest processing conditions such as disinfection, drying, grinding, packaging, and storage as well as environmental factors such as quality of soil, use of fertilizers, and climate change could impact the yield and stability of curcuminoids, which are known to be sensitive to oxygen, sunlight, and pH [6]. Even Ayurveda reports different uses for fresh and dried curcuma [7]. Turmeric is processed differently in many countries; in Brazil, for example, the rhizomes are dried in sunlight prior to grinding, while in India, the rhizomes are boiled in water under alkaline conditions [6]. These processing techniques affect curcuminoids concentration since heat treatment resulted in a loss of 27–53% of curcumin content [8]. 

The drying technique could significantly impact the quantity of curcuminoids, for example, sun drying results in 36.5% degradation in curcuminoids compared to conventional oven drying, fluidized bed drying, or freeze drying, which barely affect curcuminoids concentration [9,10,11,12]. Globally, turmeric is commercialized in the form of fresh or dried powders with an overall production exceeding 37,000 tons [9]. Even the packaging material could affect curcuminoid content in turmeric, where aluminium bags showed better capacity of preventing the decomposition of curcuminoids (up to 14%) in turmeric powder exposed to UV radiation compared to other packaging material [9]. Disinfection of turmeric powders using γ-radiation under air was reported to be the best in combating microbes without affecting curcuminoid stability. In China, herbs and spices are processed in different adjuvants such as honey or vinegar. It was reported that processing of curcuma in vinegar enhanced its anti-angiogenic activity in vivo [13]. Curcuminoids concentration was not significantly different when turmeric was stored at room temperature (27 °C) or under refrigerated conditions (4 °C) [14]. 

Considering the health benefits of turmeric and its global use in the food industry and in view of the lack of comprehensive data on the impact of postharvest conditions on curcuminoids content in turmeric essential oil, we investigated the influence of processing (fresh vs. dried milled rhizomes) and extraction techniques (hot hydrodistillation vs. cold hexane extraction) on the yield of bioactives (including curcuminoids) in turmeric essential oil using GC-MS analysis. We have likewise investigated the impact of processing on the antioxidant and enzyme inhibitory capacities of turmeric oil where the correlation between their chemical content and the biological activities was investigated using multivariate analysis and supported by in silico studies and molecular dynamics.

## 2. Results and Discussion

### 2.1. GC-MS Analysis of Curcuma Essential Oils in Fresh and Dried Samples Obtained by Hydrodistillation and Hexane Extraction

GC-MS investigations were performed on the essential oils obtained from fresh and dried rhizomes of *Curcuma longa* L. using hydrodistillation and *n*-hexane extraction. The dried rhizomes were obtained from commercial stores to mimic what is consumed by consumers. A total of 95.6%, 86.5%, 96.9%, and 90.4% of the essential oil components were identified in fresh hydrodistilled, fresh hexane-extracted, dried hydrodistilled, and dried hexane-extracted rhizome samples, respectively (Table 1). A list of the major compounds existing in the four turmeric samples is presented in the Supplementary Materials. In the fresh essential oil samples, tumerone and curlone were the major components. Meanwhile, ar-turmerone was not detected in the tested fresh turmeric samples, although it was observed in fresh turmeric grown in Bangladesh [15] and India [16]. Oxygenated sesquiterpenes constituted ca. 83.8% and 71.9% of the GC-MS chromatograms of the fresh hydrodistilled and fresh hexane-extracted turmeric essential oils, respectively.

The essential oils obtained from the dried turmeric displayed different chromatographic behavior based on the technique used. The hydrodistillation afforded D-limonene, ar-turmerone, and tumerone as major constituents, however the hexane extraction yielded ar-turmerone, curlone, and β-sesquiphellandrene as majors. Dried samples were predominated with oxygenated sesquiterpenes—but to a lesser extent than those present in fresh samples—reaching up to 48.8% and 42.4% in the hydrodistilled and hexane-extracted essential oils, respectively. Although tumerone was the predominant component in the fresh hydrodistilled and fresh hexane-extracted essential oils, making up to 60.8% and 51.6% in both samples, respectively, the dried samples showed different behavior, with D-limonene being the major component in the dried hydrodistilled essential oil (23.2%) and ar-turmerone being the predominant compound in the dried hexane-extracted essential oil (26.2%). Surprisingly, D-limonene was never detected in the dried, hexane-extracted turmeric sample, not even at a trace level, but ar-turmerone was observed at lower concentrations in the dried hydrodistilled essential oil (16.2%).

**Figure 1:**
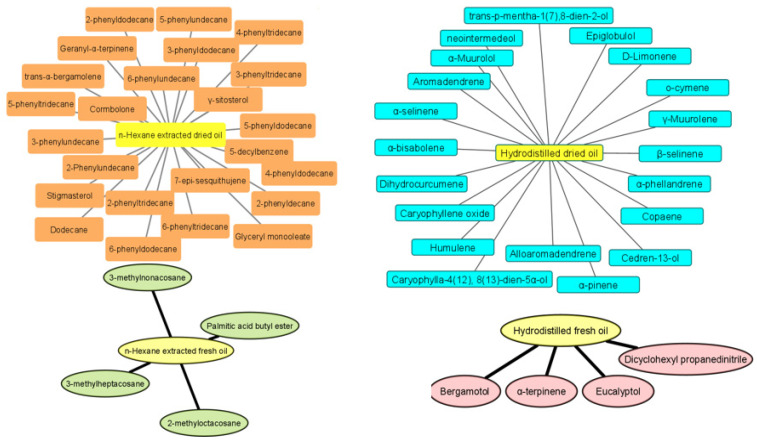
Components that are detected exclusively in the fresh hydrodistilled essential oil (pink), fresh essential oil extracted by *n*-hexane (green), hydrodistilled essential oil from dried turmeric rhizomes (turquoise), and essential oil from dried sample extracted by *n*-hexane (orange).

Some components were exclusively found in the fresh hydrodistilled essential oil, while others were only detected in the fresh essential oil extracted by *n*-hexane (Figure 1). Similarly, certain compounds were observed either in the hydrodistilled or in the hexane-extracted essential oils from dried turmeric rhizomes (Figure 1). It was found that terpinolene and turmeric were present only in fresh hydrodistilled turmeric samples at far higher concentrations, while aromatic turmeric and palmitic acid monoglyceride were only detectable in the essential oils from dried turmeric. Although β-curcumene was detected only in the fresh samples, its α-isomer was present in all samples except for the hydrodistilled fresh essential oil. Some components were detected only in those essential oils obtained by hydrodistillation such as α-terpineol, *trans*-longipinocarveol (its concentration is much higher in the hydrodistilled essential oil of dried curcuma rhizomes than the fresh ones), and zingiberenol, while others were found exclusively in the essential oils extracted by *n*-hexane such as β-farnesene (the only metabolite so far). Compounds detected in all turmeric samples, whether fresh or dried and regardless of the processing step (i.e., hydrodistillation or hexane extraction) were caryophyllene, β-bisabolene, β-sesquiphellandrene, curlone, (6*R*, 7*R*)-bisabolone, and *E*-atlantone.

To summarize, the fresh turmeric samples contain the sesquiterpene ketone tumerone as the major component in the hydrodistilled and hexane-extracted essential oil, constituting ca. 60.8% and 51.6%, respectively. The closely related compound curlone represented ca. 15.6% (in hydrodistilled essential oil) and 17.0% (in hexane-extracted essential oil) of the total GC-MS chromatogram based on their relative peak areas. Caryophylla-4(12), 8(13)-dien-5α-ol, and neo-intermedeol were exclusively detected in the hydrodistilled essential oil obtained from fresh rhizomes. Similarly, terpinolene was present in the hydrodistilled essential oil in concentrations up to 4.4% but in negligible quantities in the hexane-derived essential oil (0.43%). Zingiberene and β-sesquiphellandrene were primarily concentrated in the hexane-extracted essential oils. They were 4x their concentration in the hydrodistilled essential oil (zingiberene: 1.5%—hydrodistilled, 5.9%—hexane; β-sesquiphellandrene: 1.0%—hydrodistilled, 4.2%—hexane). 

In the dried samples, the β-sesquiphellandrene concentration increased, becoming 3x as predominant in the hexane-derived essential oil (2.3%—hydrodistilled; 6.7%—hexane). Tumerone, curlone, and zingiberene concentrations were significantly reduced, where tumerone (6.0%) was exclusively detected in the hydrodistilled essential oil while zingiberene (3.7%) was solely observed in the hexane-derived essential oil. Curlone concentration was doubled in the hexane-extracted essential oil (5.7%—hydrodistilled and 10.4%—hexane). Components such as D-limonene, caryophyllene, ar-turmerone, α-curcumene, and caryophyllene oxide were solely detected in the dried samples and were never observed in the fresh essential oil. D-limonene constituted ca. 23.2% while caryophyllene oxide reached ca. 5.7% of the dried essential oil, and both of them were only identified in the hydrodistilled essential oil sample. Caryophyllene was almost exclusively present in the hydrodistilled essential oil with only negligible amounts in the hexane-extracted essential oil (4.5%—hydrodistilled; 0.16%—hexane). The aromatic compounds ar-Turmerone and α-curcumene were 1.6× concentrated in the hexane-extracted essential oil.

### 2.2. Assessment of the In Vitro Antioxidant Properties of Turmeric Essential Oil Using DPPH, ABTS, CUPRAC, FRAP, Phosphomolybdenum, and Ferrozine Assays

The antioxidant assays measure the potential of the substance to reduce the effects of oxidative stress. With this in mind, scientists are searching for new effective alternatives with better safety profiles as those obtained from natural sources. The antioxidant effectiveness of turmeric essential oil samples was investigated using DPPH and ABTS in vitro assays (Table 2). The hexane-extracted fresh turmeric showed the highest activity (DPPH: 23.53 mg TE/g; ABTS: 66.24 mg TE/g). Generally, the hexane-extracted essential oils were more active than the hydrodistilled ones; however, the hydrodistilled essential oil from fresh samples showed no activity towards DPPH. The hydrodistilled essential oil of dried samples was found to be the weakest in the ABTS assay (17.58 mg TE/g). The superior antioxidant power of hexane-extracted essential oils may be attributed to the presence of zingiberene and β-sesquiphellandrene, which were present in higher concentrations (ca. 4× more concentrated) in the hexane extract. Consistent with our findings, these compounds have been described as powerful antioxidants in previous studies [17].

Reduction ability is associated with the electron donating ability of antioxidant compounds, and high reduction potential indicates high antioxidant properties. For this purpose, CUPRAC and FRAP assays were performed. The hexane-extracted essential oil of fresh turmeric displayed the highest reducing power (CUPRAC: 172.49 mg TE/g; FRAP: 103.40 mg TE/g), followed by the essential oils from hexane extract of dried turmeric (CUPRAC: 161.14; FRAP: 70.20 mg TE/g), and the hydrodistilled essential oils of fresh (CUPRAC: 112.35 mg TE/g; FRAP: 53.11 mg TE/g) and dried (CUPRAC: 37.97 mg TE/g; FRAP: 34.75 mg TE/g) samples.

The phosphomolybdenum assay is based on the reduction of Mo (VI) to Mo (V) by antioxidant compounds at acidic pH. As can be seen in Table 2, the reducing power of fresh turmeric samples was higher than those of dried samples. The highest ability was found in the hydrodistilled essential oil of a fresh sample with 15.36 mmol TE/g and could be explained by the presence of compounds such as terpinolene, caryophylla-4(12),8(13)-dien-5-α-ol, and neo-intermedeol.

The chelation of transition metals is associated with the management of hydroxy radicals production in the Fenton reaction, therefore the metal chelation assessment (MCA) of turmeric samples were investigated using ferrozine assay. Similar to the results obtained from the phosphomolybdenum assay, the hydrodistilled essential oil from fresh samples showed the strongest chelating ability with 28.91 mg EDTAE/g, however the results were not statistically different from hexane-extracted essential oil of the dried sample. The hydrodistilled essential oil of the dried sample displayed the weakest metal chelation power with 6.65 mg EDTAE/g. 

Altogether, the antioxidant properties of turmeric depend on whether the samples are dried or fresh. Based on the obtained results, we recommend the use of fresh rhizomes for nutritional or medical purposes due to their higher antioxidant power.

### 2.3. Assessment of the Enzyme Inhibitory Potential of Turmeric-Derived Essential Oils

Enzymes are cornerstones in manipulating several disorders such as Alzheimer’s disease, diabetes, and obesity [18]. Therefore, the enzyme inhibitory properties of turmeric samples against critical enzymes such as cholinesterase (involved in Alzheimer’s), amylase, glucosidase (involved in carbohydrates digestion and diabetes), and tyrosinase (key enzyme in the synthesis of melanin and is involved in hyperpigmentation disorders) were tested (Table 3). The hydrodistilled essential oil from fresh samples exhibited the strongest inhibitory effects on acetyl and butylcholinesterases (AChE: 2.72 mg GALAE/g; BChE: 6.35 mg GALAE/g); however, weak activity was observed by the hexane-extracted essential oil of the dried sample (2.17 mg GALAE/g). This might be attributed to the presence of some metabolites in the hydrodistilled fresh essential oil such as terpinolene, which was previously reported to display anti-cholinesterase activity [19]. The hydrodistilled essential oils displayed stronger inhibitory activities against tyrosinase compared to the hexane-extracted essential oils. Generally the strength of their tyrosinase inhibitory effect is arranged as follows: hydrodistilled essential oil of dried sample > hydrodistilled of fresh sample > hexane extract of dried sample > hexane extract of fresh sample. The richness of the hydrodistilled essential oil from the dried sample in turmerone, whose previous in silico studies showed its good binding affinity to tyrosinase binding sites [20], might be the reason for its superior activity. For amylase inhibition, the most active samples were essential oils from hexane extracts of fresh (1.08 mmol ACAE/g) and dried (1.05 mmol ACAE/g) samples but no statistical differences were observed between them. Interestingly, the hydrodistilled essential oil of fresh samples exhibited ca. five times more amylase-inhibitory activity than that of hydrodistilled essential oil of dried samples. The differences could be explained by the presence of some compounds such as terpinolene and neo-intermedeol, which were only identified in the hydrodistilled essential oil of fresh samples. For glucosidase inhibition, the most active essential oil was the one hydrodistilled from fresh turmeric (2.45 mmol ACAE/g), followed by the hexane-extracted essential oil of fresh (2.26 mmol ACAE/g) and dried (2.15 mmol ACAE/g) curcuma, then finally the hydrodistilled essential oil of dried rhizomes (1.27 mmol ACAE/g). When combining the results of both the AChE and BChE, we can conclude that the fresh hydrodistilled essential oil is the best among others in managing Alzheimer’s disease. On the other hand, amylase and glucosidase results revealed that the fresh turmeric could be of potential benefit for treating diabetes compared to the dried sample.

### 2.4. Chemometric Studies Using Multivariate Analysis

**Figure 2:**
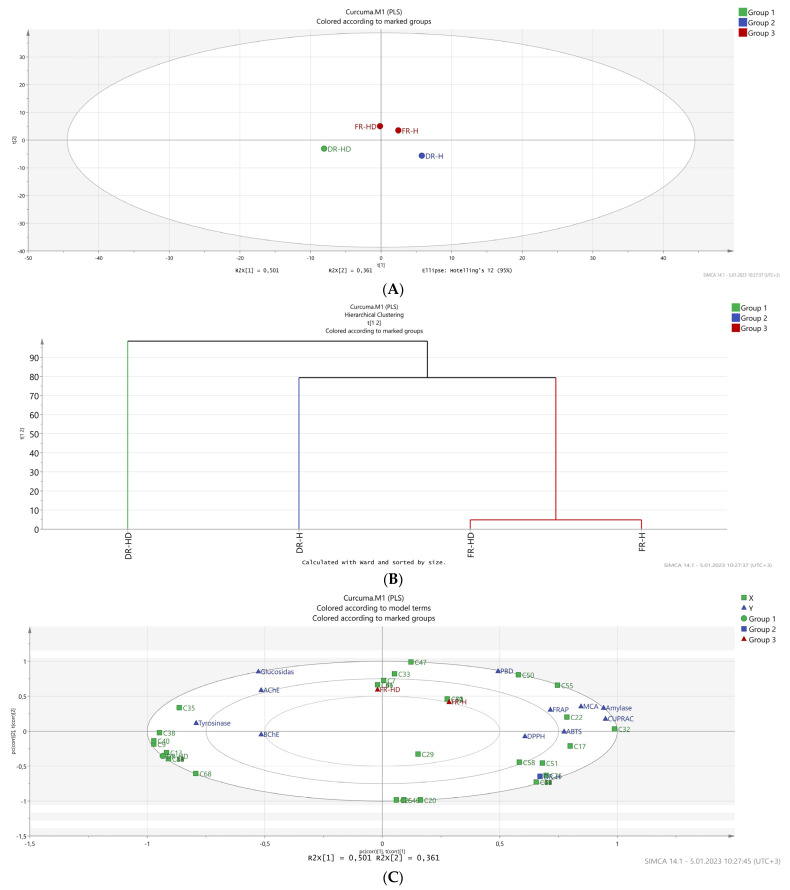
(**A**) Partial least square discriminant analysis (PLS-DA) analysis between the tested samples based on their chemical profiles and biological activities, (**B**) hierarchical cluster analysis between the tested samples, and (**C**) biplot distribution from PLS-DA analysis between the tested solvents based on chemical profiles and biological activities.

Multivariate analysis is gaining interest in the assessment of the association of different parameters. In this regard, multivariate analysis on the tested samples was performed, and the results are presented in Figure 2. A hierarchical cluster analysis was initially constructed to identify the differences between the tested samples. Samples were classified into three groups based on their chemical composition and biological activities. Both essential oils obtained from fresh turmeric were classified in the same group; however, the essential oils obtained from the dried samples were divided into different groups since the hydrodistilled and hexane-extracted essential oils from dried samples showed different chemical composition and biological activities.

Compounds such as D-tumerone, D-limonene, caryophyllene oxide, and caryophyllene (present in negligible quantities in hexane-extracted essential oil) were identified almost only in the hydrodistilled essential oil, while zingiberene was exclusively detected in the essential oil from hexane extraction. Even compounds such as curlone, β-sesquiphellandrene, and ar-turmerone were 2×, 3×, and 1.6× more concentrated, respectively, in the hexane-derived essential oil than in the hydrodistilled one. These differences in chemical composition consequently affected their biological activities. In most radical scavenging and reducing power assays, the essential oil extracted by hexane was more active than the hydrodistilled essential oil. In the fresh samples, the chemical composition was almost similar (only minor differences were observed) with tumerone and curlone being the dominant components in the hydrodistilled and hexane-derived essential oils.

### 2.5. In Silico Investigations

**Figure 3:**
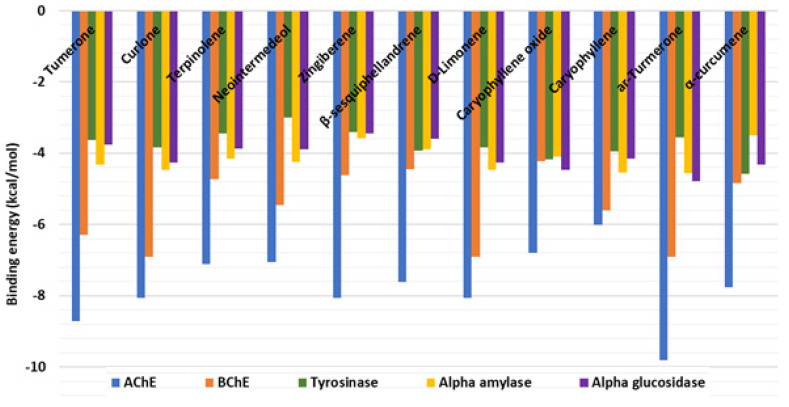
Binding energies (docking scores) of bioactive metabolites in turmeric essential oil samples.

All docked bioactive compounds were found to bind to the five enzymes, with an apparent preference for AChE and BChE based on the docking scores predicted in terms of binding energy (Figure 3). 

**Figure 4:**
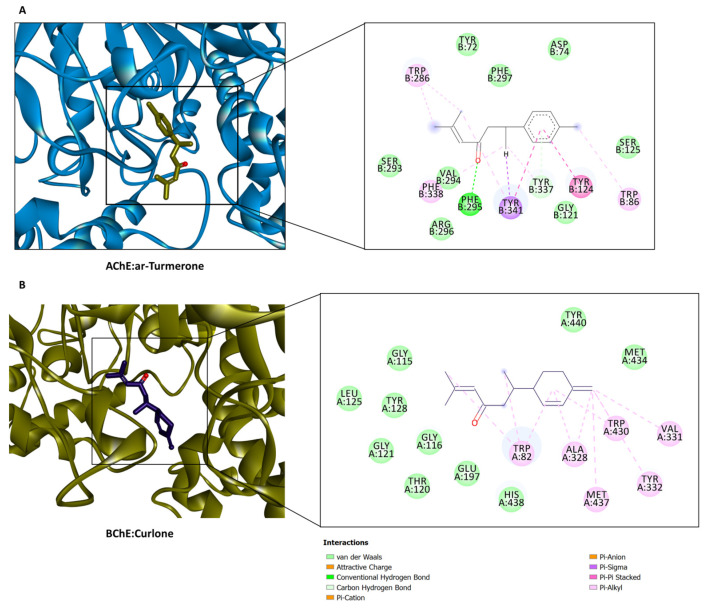
Protein–ligand interactions of (**A**) ar-turmerone with AChE, and (**B**) curlone with BChE.

**Figure 5:**
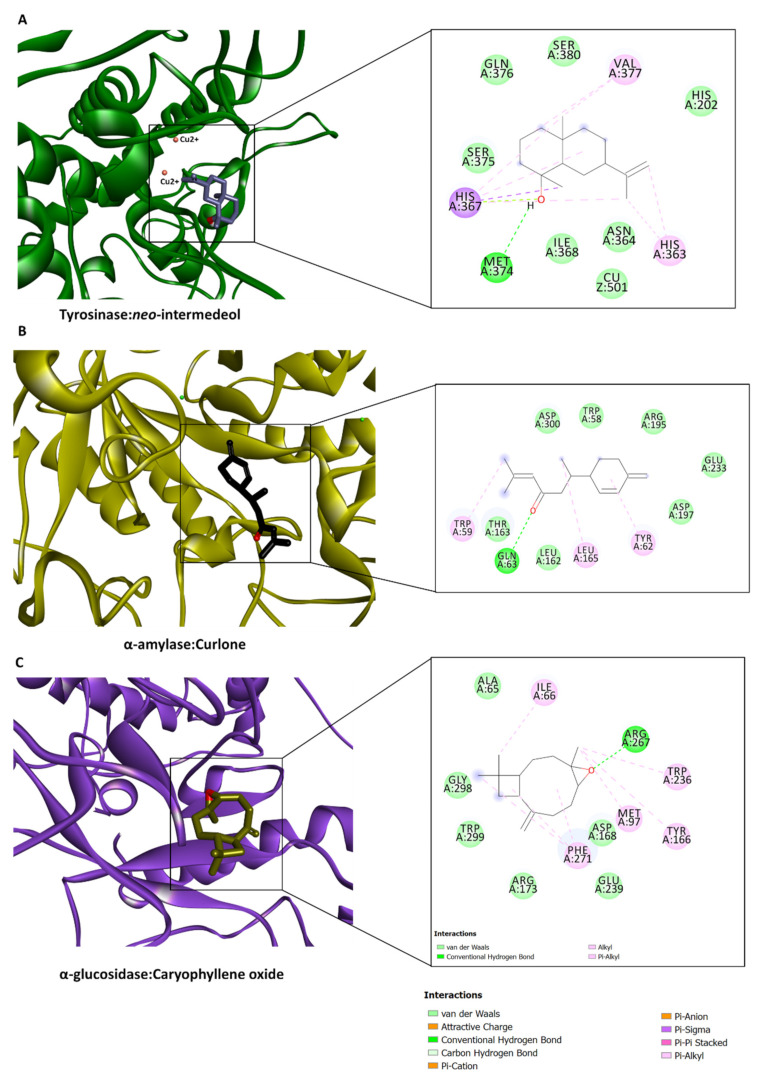
Protein–ligand interactions between (**A**) tyrosinase and neo-intermedeol, (**B**) α-amylase and curlone, and (**C**) α-glucosidase and caryophyllene oxide.

The protein–ligand interaction patterns provided insights into the binding propensity of some selected compounds. For example, ar-turmerone occupied the catalytic channel of AChE by forming an H-bond with Phe295 through the ligand carbonyl group, a couple of hydrophobic interactions with Trp86, Trp286, Phe338, Tyr341, a π-σ interaction with Tyr341, π-π interactions with Tyr124 and Tyr341, as well as several Van der Waals interactions that reinforced the binding (Figure 4A). On the other hand, curlone, a sesquiterpenoid with a high similarity in structure to ar-turmerone, bound to the catalytic site of BChE in the opposite orientation and formed multiple hydrophobic interactions near the entrance to the channel and several Van der Waals interactions deep inside the channel (Figure 4B). Interestingly, neo-intermedeol fits in the relatively narrow active site cavity of tyrosinase, forming an H-bond with Met374 (via the hydroxyl group), a π-σ interaction with His367, multiple hydrophobic interactions, and Van der Waals interactions, however, one of the active site copper ions was only engaged in a Van der Waals interaction (Figure 5). The carbonyl group in curlone formed an H-bond with Gln63 in the α-amylase active site. Other interactions formed include hydrophobic bindings with Trp59, Ty62, and Leu165, as well as a couple of Van der Waals interactions with other active site residues (Figure 5B). Caryophyllene oxide is completely buried in the catalytic cavity of α-glucosidase, forming an H-bond with Arg267 and multiple hydrophobic as well as Van der Waals interactions all over the channel (Figure 5C). It is clear from the in silico studies that all of the major compounds showed a good binding affinity with the AChE enzyme, which explains why the in vitro AChE inhibitory activity showed a slight difference between the four studied samples (2.17–2.72 mg GALAE/g). However, the presence of turmerone, curlone, terpinolene, zingiberene, β-sesquiphellandrene, and caryophyllene in the fresh hydrodistilled essential oil representing ca. 84% of its composition might be the reason beyond its in vitro BChE inhibitory activity. Regarding the potential antidiabetic activity of the fresh curcuma essential oil, we can conclude from the in silico studies that turmerone and curlone representing ca. 76% and 68%, respectively, of the hydrodistilled and hexane-extracted essential oil and showing good binding scores act synergistically to elicit the in vitro amylase and glycosidase inhibitory activities of the fresh samples.

**Figure 6:**
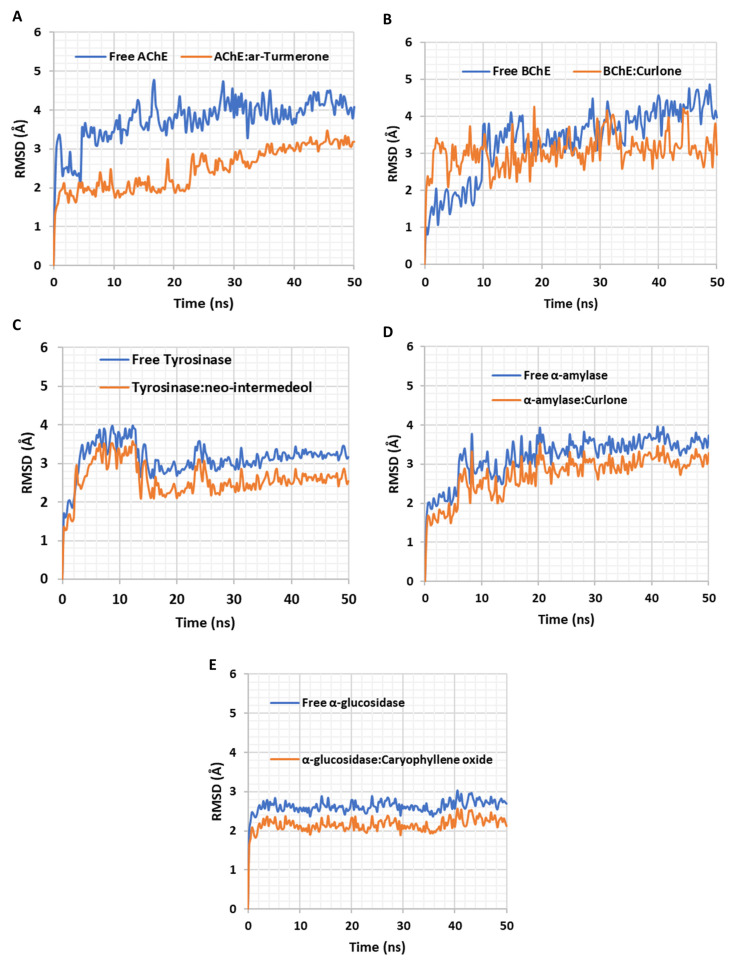
Molecular dynamics (MD) simulations. Root-mean-square displacement (RMSD) profiles of (**A**) AChE and ar-turmerone, (**B**) BchE and curlone, (**C**) tyrosinase and neo-intermedeol, (**D**) α-amylase and curlone, and (**E**) α-glucosidase and caryophyllene oxide. The RMSD variations indicate the stability of the ligand binding mode over time. The complexes displayed higher structural stability over time.

Furthermore, 50 ns long molecular dynamics (MD) simulations were carried out on AChE-turmerone, BChE-curlone, tyrosinase-neo-intermedeol, α-amylase-curlone, and α-glucosidase-caryophyllene oxide docking complexes. Analysis of root-mean-square displacement (RMSD) profiles of the complexes compared to those of the unbound proteins showed that ligand binding was associated with reduced structural variations (Figure 6), indicating the stability of the ligand binding mode over time.

Taken together, hydrophobic interactions and H-bondings are the key associations through which the selected bioactive compounds in turmeric essential oil samples bind to the enzymes. These compounds displayed potential binding mode stability over 50 ns of MD simulation. 

## 3. Materials and Methods

### 3.1. Plant Collection

Fresh *Curcuma longa* rhizomes were collected from the Faculty of Pharmacy, Ain Shams University botanical garden (Cairo, Egypt). The dried rhizomes were purchased from a local market (Cairo, Egypt). The dried and fresh samples were sliced into small pieces prior to analysis and stored at −20 °C. Voucher specimens were kept at the Department of Pharmacognosy, Faculty of Pharmacy, Ain Shams University and given the codes PHG-P-CL-424 and PHG-P-CL-425.

### 3.2. Processing of Turmeric Samples

Fresh and dried curcuma rhizomes weighing 200 g each, were washed, sliced, and separately hydrodistilled using distilled water on a Clevenger system for 4 h. At the end of the distillation process, the yellow-colored essential oils were collected, weighed, and dried over anhydrous sodium sulfate. Their yield after hydrodistillation was 0.25% for the fresh sample and 2.3% for the dried one. 

For the extraction process, 20 g of fresh and dried sliced curcuma samples were macerated in distilled analytical grade *n*-hexane (Nasr Pharmaceuticals, Cairo, Egypt) for 48 h separately. The extracts were filtered and concentrated under reduced pressure at 45 °C. The essential oily residues (0.54% and 0.6% for the fresh and dried samples, respectively) were stored in amber, air-tight sealed vials at −20 °C until further analysis. The yield was calculated as per 100 g fresh and dried curcuma and expressed in % (*w*/*w*). 

### 3.3. Assessment of the Chemical Content of Turmeric Essential Oils Using GC-MS Analysis

The GC-MS analysis was performed using a Shimadzu GCMS-QP 2010 (Kyoto, Japan). For the essential oil samples, the oven temperature was set and maintained at 45 °C for 2 min and supplied with DB-5 capillary column 30 m in length, internal diameter of 0.25 mm, and film thickness of 0.25 μm, Restek, PA, USA. Injection temperature was set at 280 °C. The temperature program was set as follows: initial temperature at 45 °C for 2 min, temperature was then raised to 300 °C at a rate of 5 °C/min and maintained at 300 °C for 5 min. Helium with a flow rate of 1.37 mL/min was selected as an inert carrier gas. Samples (5% *v*/*v*) were injected with a split ratio 30:1, and injection volume was 1 μL. The ion source temperature was set at 220 °C and the interface temperature at 280 °C. The electron impact ionization (EI) was 70 eV and the mass spectra were analyzed in the scan mode over the range of 35 to 500 amu. The hexane samples were run under the same conditions, except for the oven temperature, which was set and maintained at 50 °C for 3 min, temperature was raised to 300 °C at a rate of 5 °C/min and maintained at 300 °C for 10 min [21,22]. The identification of the essential oil components was based on the comparison of their mass fragmentation data (MS) and their Kovats indices (KI) with those present in the NIST Mass Spectral Library (2011), the 4th edition of “Identification of Essential oil Components by Gas Chromatography/Quadrupole Mass Spectroscopy” [23], *Wiley Registry of Mass Spectral Data* 8th edition, and those data reported in the literature [24,25,26,27].

### 3.4. In Vitro Antioxidant Analysis

The antioxidant activity of the tested samples was determined in triplicate according to previously described methods [28,29]. DPPH and ABTS radical scavenging activity, cupric ion reducing antioxidant capacity (CUPRAC), and ferric ion reducing antioxidant power (FRAP) were expressed as mg trolox equivalents (TE)/g essential oil. The metal chelating ability (MCA) was reported as mg EDTA equivalents (EDTAE)/g essential oil, whereas the total antioxidant activity (phosphomolybdenum assay, PBD) was expressed as mmol TE/g extract. All experimental details are given in the Supplementary Materials. 

### 3.5. Enzyme Inhibitory Analysis

The enzyme inhibitory properties of the tested essential oils were investigated against AChE, BChE, tyrosinase, amylase, and glucosidase. The activities were determined in triplicate as reported by our previous methods [28,29]. AChE and BChE inhibitory activities were given as mg galanthamine equivalents (GALAE)/g essential oil, tyrosinase inhibitory activity was expressed as mg kojic acid equivalents (KAE)/g extract, amylase and glucosidase inhibitory activities were presented as mmol acarbose equivalents (ACAE)/g essential oil. All experimental details are given in the Supplementary Materials. 

### 3.6. Molecular Modeling and Dynamics Studies

The crystal structures of AChE (PDB ID: 6O52) [30], BChE (PDB ID: 6EQP) [31], and α-amylase (PDB ID: 1B2Y) [32] were retrieved from the protein data bank (PDB) (https://www.rcsb.org/) (accessed on 6 October 2022). Since the crystal structures of human tyrosinase and α-glucosidase have not yet been resolved, those of *Priestia megaterium* tyrosinase (6QXD) [33] and *Mus musculus* α-glucosidase (7KBJ) [34] were retrieved to serve as templates for building their human models using respective UniProt sequences P14679 and P0DUB6. The details of the homology modeling have been given in our previous work [35]. All the crystal structures and the built models were prepared. The pK_a_ of the titratable residues in each protein was predicted and was used to protonate the proteins at the physiological pH of 7.4 using the Playmolecule ProteinPrepare module (https://playmolecule.com/proteinPrepare/) (accessed on 10 December 2022) [36].

The ligand 3D structures were downloaded from the PubChem database (https://pubchem.ncbi.nlm.nih.gov/) (accessed on 5 December 2022). Geometry optimization was done using Biovia Discovery Studio Visualizer v4.5 (Dassault Systèmes Biovia Software Inc, San Diego, CA, USA, 2012). The co-crystal ligand in each complex was used to define the docking grid box dimension and coordinates using AutoDockTools 1.5.6, and each ligand was docked into the binding pocket of each protein using AutoDock 4.2.6 (https://autodock.scripts.edu) (accessed on 10 December 2022) [37]. The protocol employed in previous docking simulations was likewise here adopted [38]. The docking (binding energy) scores were calculated, and protein–ligand interactions were analyzed using Biovia Discovery Studio Visualizer v4.5 (Dassault Systèmes Biovia Software Inc, San Diego, CA, USA, 2012). Finally, some selected docking complexes were submitted to 50 ns production molecular dynamics simulations to examine the stability of the ligand binding mode using NAMD software (http://www.ks.uiuc.edu/Research/namd/) (accessed on 10 December 2022) [39]. The details of the simulations including the used solvent model, the added ions, the performed minimization and equilibration, as well as the ligand parameterization was done as previously described [40,41].

### 3.7. Statistical Analysis

Data are presented as mean ± standard deviation of three (n = 3) replicates. One-way analysis of variance with Tukey’s post hoc test was conducted where *p* < 0.05 was considered as statistically significant. The statistical evaluation was performed using Graphpad version 9.0. The relationship between molecules and their antioxidant/enzyme inhibitory activities was assessed by multivariate analysis. Partial least squares discriminant analysis (PLS-DA) was performed for comparison. We used the percentage of volatile compounds and the mean of biological activity assays. The statistical analysis was done using SIMCA 14.0.

## 4. Conclusions

The GC-MS investigations on curcuma rhizomes under different processing steps and extraction techniques showed a difference in the chemical profile between fresh and dried rhizomes. Differences in the extraction techniques affected mainly the concentrations of the minor constituents. The variations in the essential oil metabolic profile have consequently affected its antioxidant properties. In the ABTS, CUPRAC, and FRAP assays, the fresh hexane-extracted essential oil samples were generally more active than the dried ones in reducing oxidative stress. For the acetylcholinesterase, α-amylase, and α-glucosidase inhibitory activities, the fresh samples were more active than the dried ones, however no significant variations were observed between the hydrodistilled and hexane-extracted essential oils. The superiority of the activity of the fresh turmeric samples might be attributed to their high concentration of tumerone and curlone compared to the dried turmeric. The docking performed with the 11 major metabolites, namely tumerone, curlone, terpinolene, neo-intermedeol, zingiberene, β-sesquiphellandrene, D-limonene, caryophyllene oxide, caryophyllene, ar-turmerone, and α-curcumene, on the five different enzyme targets revealed that hydrophobic interactions and hydrogen bindings were the key characteristic attachments between the bioactive compounds and the target enzymes with binding stability over 50 ns of molecular dynamics simulations as revealed from the RMSD variations. Curlone fits quite well in the pocket of butylcholinesterase and α-amylase, while neo-intermedol, caryophyllene oxide, and ar-turmerone showed high binding scores on tyrosinase, α-glucosidase, and acetylcholinesterase, respectively.

## Figures and Tables

**Table 1:** GC-MS analysis of the volatile components in the essential oil of the fresh and dried curcuma samples obtained by hydrodistillation and by *n*-hexane extraction.

No.	t_R_ (min)	Compound Name	Molecular Formula	KI_exp_^a^	KI_rep_^b^	Peak Area (%) in Fresh Curcuma		Peak Area (%) in Dried Curcuma		Chemical Class
						Hydrodistilled	Hexane- Extracted	Hydrodistilled	Hexane- Extracted	
1.	7.16	α-Pinene	C_10_H_16_	931	931	-	-	0.62	-	Monoterpene hydrocarbon
2.	9.28	α-Phellandrene		1003	1003	-	-	1.58	-	
3.	9.67	α-Terpinene		1016	1016	0.18	-	-	-	
4.	9.91	o-Cymene	C_10_H_14_	1023	1023	-	-	2.26	-	
5.	10.05	D-Limonene	C_10_H_16_	1028	1028	-	-	23.21	-	
6.	10.10	Eucalyptol	C_10_H_18_O	1029	1029	1.92	-	-	-	Monoterpene ether
7.	11.91	Terpinolene	C_10_H_16_	1088	1088	4.47	0.43	-	-	Monoterpene hydrocarbon
8.	14.98	*trans*-*p*-Mentha-1(7),8-dien-2-ol	C_10_H_16_O	1188	1185	-	-	0.33	-	Monoterpene alcohol
9.	15.07	α-Terpineol	C_10_H_18_O	1191	1191	0.17	-	0.51	-	
10.	15.50	Dodecane	C_12_H_26_	1189	1200	-	-	-	0.08	Aliphatic hydrocarbon
11.	20.43	Copaene	C_15_H_24_	1378	1378	-	-	0.33	-	Sesquiterpene hydrocarbon
12.	21.47	7-*epi*-Sesquithujene		1396	1391	-	-	-	0.08	
13.	21.64	Caryophyllene		1423	1423	0.35	0.97	4.58	0.16	
14.	22.30	trans-α-Bergamotene		1428	1428	-	-	-	0.04	
15.	22.15	Aromandendrene		1443	1441	-	-	0.20	-	
16.	22.54	Humulene		1458	1458	-	-	0.62	-	
17.	22.54	β-Farnesene		1458	1458	-	0.25	-	0.24	
18.	22.74	Alloaromadendrene		1465	1465	-	-	0.20	-	
19.	23.11	γ-Muurolene		1479	1479	-	-	0.21	-	
20.	23.25	α-Curcumene	C_15_H_22_	1485	1485	-	0.77	2.32	3.73	
21.	23.41	β-Selinene	C_15_H_24_	1491	1491	-	-	2.36	-	
22.	23.58	Zingiberene		1497	1497	1.57	5.90	-	3.73	
23.	23.63	α-Selinene		1499	1499	-	-	2.82	-	
24.	23.75	α-Bisabolene		1504	1504	-	-	0.53	-	
25.	23.93	β-Bisabolene		1511	1511	0.15	0.57	1.11	1.41	
26.	24.31	β-Sesquiphellandrene		1527	1527	1.04	4.28	2.31	6.74	
27.	24.88	5-Decylbenzene	C_16_H_26_	1529	1535	-	-	-	0.64	
28.	25.27	Epiglobulol	C_15_H_26_O	1565	1564	-	-	0.48	-	Sesquiterpene alcohol
29.	25.72	aR-Turmerol (Bisacumol)	C_15_H_22_O	1584	1584	1.28	-	0.72	1.33	
30.	25.77	2-Phenyl-decane	C_16_H_26_	1589	1588	-	-	-	1.26	Aromatic hydrocarbon
31.	25.87	Caryophyllene oxide	C_15_H_24_O	1590	1590	-	-	5.74	-	Sesquiterpene ether
32.	25.96	*trans*-Sesquisabinene hydrate	C_15_H_26_O	1593	1590	0.47	0.49	-	0.71	Sesquiterpene alcohol
33.	26.31	β-Curcumene	C_15_H_22_	1608	1517	1.76	0.44	-	-	Sesquiterpene hydrocarbon
34.	26.35	Dihydrocurcumene	C_15_H_24_	1609	1692	-	-	1.67	-	
35.	26.54	*cis*-Sesquisabinene hydrate	C_15_H_26_O	1618	1620	0.72	0.94	1.33	-	Sesquiterpene alcohol
36.	26.72	6-Phenylundecane	C_17_H_28_	1621	1628	-	-	-	1.50	Aromatic hydrocarbon
37.	26.81	5-Phenylundecane		1625	1633	-	-	-	2.19	
38.	26.96	Zingiberenol	C_15_H_26_O	1636	1620	0.67	-	1.18	-	Sesquiterpene alcohol
39.	27.05	4-Phenylundecane	C_17_H_28_	1635	1643		-	-	2.55	Aromatic hydrocarbon
40.	27.06	*trans*-Longi pinocarveol	C_15_H_24_O	1640	1634	0.49	-	1.21	-	Sesquiterpene alcohol
41.	27.07	Bergamotol		1640	1657	0.73	-	-	-	
42.	27.16	Caryophylla-4(12),8(13)-dien-5-α-ol		1644	1640	-	-	3.82	-	
43.	27.37	α-Muurolol	C_15_H_26_O	1653	1653	-	-	0.71	-	
44.	27.53	3-Phenylundecane	C_17_H_28_	1656	1667	-	-		2.16	Aromatic hydrocarbon
45.	27.59	neo-intermedeol	C_15_H_26_O	1662	1660	-	-	3.28	-	Sesquiterpene alcohol
46.	27.79	ar-Turmerone	C_15_H_20_O	1671	1672	-	-	16.27	26.24	Sesquiterpene ketone
47.	28.01	Tumerone	C_15_H_22_O	1680	1680	60.80	51.65	6.07	-	
48.	28.30	Cedren-13-ol	C_15_H_24_O	1693	1690	-		1.48	-	Sesquiterpene alcohol
49.	28.88	2-Phenylundecane	C_17_H_28_	1695	1703	-	-	-	3.50	Aromatic hydrocarbon
50.	29.00	Curlone	C_15_H_22_O	1709	1701	15.61	17.00	5.72	10.40	Sesquiterpene ketone
51.	29.27	α-Atlantone		1712	1722	0.25	-	-	0.57	
52.	29.46	6-Phenyldodecane	C_18_H_30_	1720	1726	-	-	-	1.45	Aromatic hydrocarbon
53.	29.57	5-Phenyldodecane		1724	1730	-	-	-	1.55	
54.	29.84	4-Phenyldodecane		1736	1742	-	-	-	1.41	
55.	29.99	(6*R*,7*R*)-Bisabolone	C_15_H_24_O	1748	1747	1.56	1.49	0.32	1.16	Sesquiterpene ketone
56.	30.07	Dicyclohexyl-propanedinitrile	C_15_H_22_N_2_	1766	1769	0.55	-	-	-	Nitrile
57.	30.32	3-Phenyldodecane	C_18_H_30_	1757	1755 *	-	-	-	2.12	Aromatic hydrocarbon
58.	30.62	*E*-Atlantone	C_15_H_22_O	1774	1773	1.24	0.37	0.56	1.92	Sesquiterpene ketone
59.	31.17	2-Phenyldodecane	C_18_H_30_	1794	1791 *	-	-	-	3.08	Aromatic hydrocarbon
60.	31.62	6-phenyltridecane	C_19_H_32_	1815	1819	-	-	-	1.82	
61.	31.76	5-phenyltridecane		1822	1821 *	-	-	-	1.13	
62.	32.04	4-phenyltridecane		1835	1840	-	-	-	1.08	
63.	32.53	3-phenyltridecane		1859	1856 *	-	-	-	1.25	
64.	33.18	Corymbolone	C_15_H_24_O_2_	1890	1898	-	-	-	0.16	Sesquiterpene ketone
65.	33.25	Geranyl-α-terpinene	C_20_H_32_	1939	1952	-	-	-	0.11	Diterpene hydrocarbon
66.	33.36	2-Phenyltridecane	C_19_H_32_	1898	1916	-	-	-	1.95	Aromatic hydrocarbon
67.	38.36	Palmitic acid butyl ester	C_20_H_40_O_2_	2186	2188	-	0.40	-	-	Fatty acid ester
68.	44.31	Palmitic acid β-monoglyceride	C_19_H_38_O_4_	2497	2498	-	-	0.33	0.08	Glyceryl ester
69.	47.17	Glyceryl monooleate	C_21_H_40_O_4_	2682	2714	-	-	-	0.10	
70.	47.86	3-Methyl heptacosane	C_28_H_58_	2771	2771	-	0.14	-	-	Aliphatic hydrocarbon
71.	49.12	2-Methyloctacosane	C_29_H_60_	2860	2859	-	0.19	-	-	
72.	50.67	3-Methylnonacosane	C_30_H_62_	2971	2972	-	0.27	-	-	
73.	55.54	Stigmasterol	C_29_H_48_O	3220	3170	-	-	-	0.19	Sterol
74.	56.55	γ-Sitosterol	C_29_H_50_O	3285	3290	-	-	-	0.64	
Monoterpene hydrocarbons (%)						4.65	0.43	27.67	-	
Oxygenated monoterpenes (%)						2.09	-	0.84	-	
Sesquiterpene hydrocarbons (%)						4.87	13.18	19.26	16.13	
Oxygenated sesquiterpenes (%)						83.82	71.94	48.89	42.49	
Diterpene hydrocarbon (%)						-	-	-	0.11	
Others (%)						0.73	1	0.33	31.73	
Total identified (%)						95.98	86.55	96.99	90.46	

^a^ Kovats index determined experimentally on RTX-5 column relative to C8–C30 series of n-alkanes. ^b^ Reported Kovats indices (KI). t_R_ is the observed retention time for each compound. * refers to the KI reported on DB-1 capillary column. Identification of compounds was based on the comparison of their mass fragmentation data (MS) and their Kovats indices (KI) with those present in the NIST Mass Spectral Library (2011), *Wiley Registry of Mass Spectral Data* 8th edition, and those data reported in the literature.

**Table 2:** The antioxidant properties of the essential oil obtained from fresh and dried turmeric rhizomes.

Samples	Methods	DPPH (mg TE/g)	ABTS (mg TE/g)	CUPRAC (mg TE/g)	FRAP (mg TE/g)	MCA (mg EDTAE/g)	PBD (mmol TE/g)
Dried	Hydrodistilled	3.63 ± 0.59 ^c^	17.58 ± 0.77 ^d^	37.97 ± 0.99 ^d^	34.75 ± 0.82 ^d^	6.65 ± 0.26 ^c^	3.44 ± 0.09 ^d^
	hexane	15.09 ± 0.51 ^b^	52.93 ± 0.85 ^b^	161.14 ± 3.39 ^b^	70.20 ± 1.47 ^b^	26.88 ± 0.50 ^a^	7.82 ± 0.27 ^c^
Fresh	Hydrodistilled	na	22.25 ± 0.73 ^c^	112.35 ± 2.10 ^c^	53.11 ± 0.69 ^c^	28.91 ± 2.23 ^a^	15.36 ± 0.61 ^a^
	hexane	23.53 ± 0.74 ^a^	66.24 ± 0.50 ^a^	172.49 ± 3.63 ^a^	103.40 ± 2.51 ^a^	22.05 ± 1.23 ^b^	12.83 ± 0.40 ^b^
*p* value		0.0001	0.0001	0.0001	0.0001	0.0001	0.0001

Values are reported as mean ± SD of three parallel measurements. TE: trolox equivalent; EDTAE: EDTA equivalent; na: not active. Different letters indicate significant differences between tested samples (*p* < 0.05).

**Table 3:** The enzyme inhibitory properties of the essential oil obtained from fresh and dried turmeric rhizomes.

Samples	Processing	AChE (mg GALAE/g)	BChE (mg GALAE/g)	Tyrosinase (mg KAE/g)	Amylase (mmol ACAE/g)	Glucosidase (mmol ACAE/g)
Dried	Hydrodistilled	2.46 ± 0.02 ^b^	5.27 ± 0.73 ^ab^	51.54 ± 0.87 ^a^	0.19 ± 0.01 ^c^	1.27 ± 0.01 ^d^
	Hexane	2.17 ± 0.01 ^c^	4.82 ± 0.77 ^b^	22.73 ± 0.92 ^b^	1.05 ± 0.02 ^a^	2.15 ± 0.06 ^c^
Fresh	Hydrodistilled	2.72 ± 0.09 ^a^	6.35 ± 0.04 ^a^	49.83 ± 7.60 ^a^	0.94 ± 0.01 ^b^	2.45 ± 0.04 ^a^
	Hexane	2.25 ± 0.07 ^c^	3.23 ± 0.31 ^c^	17.37 ± 1.78 ^b^	1.08 ± 0.01 ^a^	2.26 ± 0.03 ^b^
*p* value		0.0001	0.001	0.0001	0.0001	0.0001

Values are reported as mean ± SD of three parallel measurements. GALAE: galanthamine equivalent; KAE: kojic acid equivalent; ACAE: acarbose equivalent. Different letters indicate significant differences between tested samples (*p* < 0.05).

## Data Availability

No new data were analyzed in the current study and data sharing is not applicable here.

## Supplementary Materials

The following supporting information can be downloaded at: https://www.mdpi.com/article/10.3390/plants12091785/s1, Table S1: Major components present in fresh hydrodistilled, fresh hexane-extracted, dried hydrodistilled, and dried hexane-extracted turmeric oil samples.
