# Supplementary material for: Comparative GC-MS Analysis of Fresh and Dried Curcuma Essential Oils with Insights into Their Antioxidant and Enzyme Inhibitory Activities
Source: Plants (Basel). 2023 Apr 27;12(9):1785. doi: 10.3390/plants12091785 (PMC10180709; doi:10.3390/plants12091785)
Supplement: Supplementary file 1 [file plants-12-01785-s001.zip › plants-2219779-supplementary.pdf]

Supplementary Information

Major components present in fresh hydrodistilled, fresh hexane-extracted, dried hydrodistilled, and dried hexane-extracted turmeric oil samples.

Compound name	Fresh samples		Dried samples		Chemical structure
	Hydrodistilled oil	Hexane-extracted oil	Hydrodistilled oil	Hexane extracted oil	
Tumerone	60.8	51.6	6.0	-----	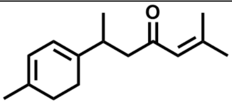
Curlone	15.6	17.0	5.7	10.4	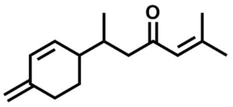
Terpinolene	4.4	0.43	-----	-----	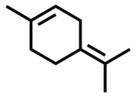
Caryophylla-4(12), 8(13)-dien-5-α-ol	3.8	-----	-----	-----	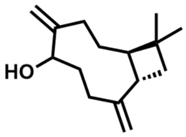
neo-intermedeol	3.2	-----	-----	-----	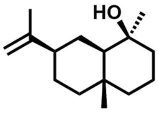
Zingiberene	1.5	5.9	-----	3.7	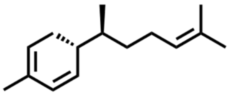
β-Sesquiphellandrene	1.0	4.2	2.3	6.7	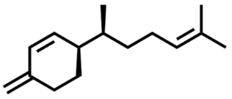
D-Limonene	-----	-----	23.2	-----	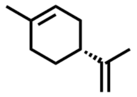
Caryophyllene oxide	-----	-----	5.7	-----	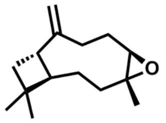
Caryophyllene	0.35	0.97	4.5	0.16	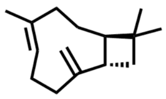
ar-Turmerone	-----	-----	16.2	26.2	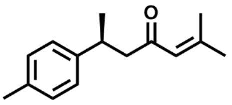
α-Curcumene	-----	0.77	2.3	3.7	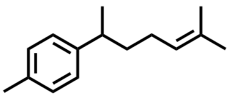
